# Artificial Intelligence in Intensive Care: From Clinical Applications to Ethical and Medico-Legal Challenges

**DOI:** 10.3390/healthcare14203382

**Published:** 2026-10-10

**Authors:** Francesco de Lazzaro, Giovanna Barletta, Eugenia Magnanimi, Bruno Cirillo, Paola Frati, Enrico Marinelli, Francesco Pugliese, Lina De Paola, Giovanni Giordano

**Affiliations:** 1 Department of Emergency, Critical Care and Trauma, University Hospital Umberto I, 00161 Rome, Italy; f.delazzaro@policlinicoumberto1.it (F.d.L.); g.barletta@policlinicoumberto1.it (G.B.); e.magnanimi@policlinicoumberto1.it (E.M.); f.pugliese@uniroma1.it (F.P.); giovanni.giordano@uniroma1.it (G.G.); 2 Emergency Department, Policlinico Umberto I, Sapienza University, 00185 Rome, Italy; bruno.cirillo@uniroma1.it; 3 Department of Anatomical, Histological, Forensic and Orthopedic Sciences, Sapienza University, 00161 Rome, Italy; paola.frati@uniroma1.it; 4 Department of Medico-Surgical Sciences and Biotechnologies, Sapienza University, 04100 Rome, Italy; enrico.marinelli@uniroma1.it; 5 Department of General, Specialized Surgery and Anesthesiology, Sapienza University, 00161 Rome, Italy

**Keywords:** artificial intelligence, clinical decision support, ethics and medico-legal issues, intensive care unit, machine learning

## Abstract

From a medico-legal perspective, AI functions as a clinical decision support tool without legal autonomy. Responsibility for AI-assisted clinical decisions remains subject to the applicable legal and regulatory framework and depends on the degree of human oversight. The use of artificial intelligence (AI) is rapidly transforming intensive care medicine, offering advanced tools for data processing, prediction, and clinical decision support. This narrative review explores current applications of AI in the intensive care unit (ICU), along with associated ethical, medico-legal, and regulatory challenges. AI systems, particularly those based on machine learning and deep learning, have demonstrated promising predictive performance across several intensive care applications. Systematic reviews of machine learning models for ICU prediction tasks have reported AUROC values generally ranging from approximately 0.80 to over 0.90 for early sepsis prediction and ARDS risk stratification, although performance decreases in external validation cohorts. AI has also shown encouraging performance in predicting weaning success and supporting mechanical ventilation management, while evidence of improved patient-centered clinical outcomes remains limited A narrative literature search was conducted in PubMed and Scopus for studies published between January 2015 and December 2025. Additionally, AI contributes to continuous patient monitoring, reduction in false alarms, and improved workflow efficiency. Despite these advances, most models are derived from retrospective and single-center datasets, limiting generalizability and real-world applicability. Moreover, only a limited number of AI systems have undergone prospective clinical evaluation or demonstrated improvements in patient-centered outcomes following real-world implementation. A key limitation of current AI systems is the “black box” nature of many algorithms, which reduces interpretability and may undermine clinician trust. Furthermore, algorithmic bias, data quality issues, and limited external validation raise concerns regarding safety, equity, and reliability. Importantly, improved predictive accuracy does not necessarily translate into better clinical outcomes, highlighting the need for prospective validation studies. From a medico-legal perspective, AI functions as a clinical decision support tool without legal autonomy. Responsibility for AI-assisted clinical decisions remains subject to the applicable legal and regulatory framework and depends on the degree of human oversight, the intended use of the AI system, and the relevant jurisdiction. The emergence of regulatory frameworks, such as the European Union Artificial Intelligence Act and the General Data Protection Regulation (GDPR), reflects the increasing regulation of healthcare AI according to the intended purpose, level of autonomy, and potential impact of each system, emphasizing requirements for transparency, human oversight, and data protection.

## 1. Introduction

Artificial intelligence (AI) encompasses computational approaches capable of analyzing large volumes of clinical data and supporting complex decision-making processes. In intensive care, the most widely adopted AI techniques are based on machine learning (ML), which identifies patterns from structured or unstructured clinical data, and deep learning (DL), a subset of ML that uses multilayer neural networks to model complex relationships. Because many DL models have limited interpretability, explainable AI (XAI) methods have been developed to improve transparency and facilitate clinicians’ understanding of algorithmic outputs [1]. Generative AI models, including transformer-based large language models (LLMs), have recently emerged as complementary tools for documentation, information synthesis, and communication support. Other AI approaches relevant to intensive care include computer vision for medical imaging, reinforcement learning for sequential treatment optimization, and explainable AI methods that improve the interpretability of model outputs. These categories are illustrative and often overlap in clinical practice. However, the present review primarily examines predictive AI models and clinical decision support systems currently applied or under evaluation in intensive care [2].

Machine learning (ML), a subfield of AI, enables these systems to learn from structured or unstructured data. The quality and representativeness of data are critical in developing AI-based decision support systems to ensure that the model is applicable to the intended population or clinical problem. Deep learning (DL) is a subset of ML based on multilayered neural networks, whose internal functioning often remains only partially interpretable—a phenomenon known as the black box problem [3]. To address this, many experts advocate for the development of “explainable AI (XAI)”, which aims to make AI model decisions transparent and understandable to human users.

Among the most widely used XAI techniques are SHAP (SHapley Additive exPlanations) and LIME (Local Interpretable Model-agnostic Explanations), which improve model interpretability by explaining the contribution of individual variables to AI predictions and facilitating clinicians’ understanding of algorithmic outputs [4].

In intensive care clinical practice, AI is currently being explored for the automation of administrative and bureaucratic processes, enhancement of diagnostic accuracy (especially in imaging), early detection of adverse events and decision support in mechanical ventilation, sedation weaning, and automated triage [5]. While these applications are promising, they raise significant ethical and legal concerns. These include the quality of training data, algorithmic transparency and explainability, data privacy, informed consent, and equity, particularly in cases where datasets do not adequately represent certain patient populations (e.g., bias related to gender, age, or race) [6].

AI systems may be high-performing but are far from infallible: bias, hallucinations, and errors remain major concerns in their clinical implementation. In the event of an algorithm-related error, questions of responsibility arise: should accountability fall on the clinician, the developer, or the institution? In response to these emerging issues, the European Union has approved the EU Artificial Intelligence Act (AIA) [7]. Under the Act, not all healthcare AI systems are uniformly classified as high risk. Rather, classification depends on the intended purpose, level of autonomy, and potential impact of the AI system. Many AI applications used for clinical decision support in intensive care are considered high risk because they may influence diagnosis or therapeutic decision-making and therefore require specific requirements for risk management, transparency, human oversight, and post-market monitoring.

The regulatory framework for data protection is supported by existing instruments such as the General Data Protection Regulation (GDPR), which covers all aspects related to the collection, use, processing, and storage of personal data [8]. Within this context, the concept of algorethics, the ethics of algorithms, is gaining traction. It calls for integrating fundamental bioethical principles (non-maleficence, beneficence, autonomy, justice) into the design, deployment, and clinical use of AI [9].

Recent reviews have primarily addressed either the technical applications of artificial intelligence in critical care or the ethical and regulatory implications of AI in healthcare. Beyond clinical decision support, artificial intelligence is increasingly influencing how patients and caregivers access and interpret health information through digital platforms and conversational AI tools. This may improve health literacy and support shared decision-making when information is accurate and evidence-based. However, the quality and reliability of AI-generated information remain critical concerns, as inaccurate or misleading content may negatively influence health perceptions and clinical decisions. These challenges highlight the importance of transparent, understandable, and human-supervised AI communication, particularly in high-risk settings such as intensive care. This narrative review aims to provide an integrated and clinically oriented perspective by examining current ICU applications alongside implementation challenges, ethical and medico-legal implications, and emerging regulatory frameworks. By combining these dimensions, we seek to bridge the gap between technological development and safe clinical implementation in intensive care practice [10,11].

## 2. Materials and Methods

This narrative review was conducted to explore the current applications, ethical implications, and regulatory challenges of artificial intelligence in intensive care medicine. Given the rapidly evolving nature of the field and the heterogeneity of study designs, a narrative approach was chosen to allow a comprehensive and critical synthesis of both technical and clinical perspectives.

### 2.1. Literature Search Strategy

A non-systematic literature search was conducted using PubMed/MEDLINE, Scopus, and Google Scholar. The search covered articles published from January 2015 to December 2025 and was limited to English-language publications because English is the predominant language of the scientific literature in this field. This may have introduced language bias by excluding relevant studies published in other languages and being limited to studies in English. The search terms included “artificial intelligence,” “intensive care unit,” “machine learning,” and “medical imaging,” yielding 210 results, as well as “artificial intelligence,” “intensive care unit,” “ethics,” and “legal,” yielding 42 results.

Representative search strategies included combinations of controlled vocabulary and free-text terms. For example, the PubMed search strategy included (“artificial intelligence” OR “machine learning” OR “deep learning”) AND (“intensive care unit” OR ICU OR “critical care”) AND (sepsis OR ARDS OR ventilation OR imaging OR ethics OR legal).

Articles were excluded if they (i) were not written in English; (ii) involved pediatric or animal populations; (iii) focused on non-ICU settings or topics unrelated to artificial intelligence in critical care; (iv) were editorials, conference abstracts, letters, or opinion papers without original data or regulatory relevance; or (v) provided insufficient methodological information to allow interpretation of their findings. Studies were selected based on their relevance to ICU applications, ethical and medico-legal issues, predictive model development or validation, and regulatory aspects of AI implementation. In total, 85 and 12 articles were analyzed for each search, respectively.

After applying the eligibility criteria and removing records considered outside the scope of this review, studies were selected for narrative synthesis based on their relevance to the topics addressed. Additional relevant references, including consensus statements and regulatory documents, were identified through manual searching. A manual search of the reference lists of relevant reviews, original articles, and regulatory documents was also performed to identify additional studies that were considered relevant but were not retrieved through the electronic database search.

### 2.2. Study Selection and Data Synthesis

Original research articles, narrative and systematic reviews, consensus documents, and regulatory reports addressing AI applications in critical care were considered. Particular emphasis was placed on studies describing real-world ICU use cases, predictive and decision support models, ethical and medico-legal considerations, and regulatory frameworks relevant to clinical implementation.

Given the narrative nature of this review and the heterogeneity of the included studies, no formal risk-of-bias assessment or quality scoring (e.g., QUADAS-2 or PROBAST) was performed. Instead, studies were critically appraised through an informal qualitative assessment based on study design, clinical relevance, data sources, validation strategy, and reported methodological limitations, including bias, interpretability, and generalizability.

### 2.3. Ethical and Regulatory Framework

Specific attention was devoted to the ethical, legal, and regulatory aspects of AI deployment in critical care, with reference to current European legislation, including the EU AIA and the GDPR. Concepts related to explainable AI, human oversight, accountability, and algorethics were integrated into the analysis to contextualize technical developments within a clinically and ethically sustainable framework.

## 3. Results

AI is increasingly being applied in various areas of the healthcare system, including robot-assisted surgery, rehabilitation, medical imaging and diagnostics, virtual patient care, medical research and drug discovery, and patient engagement and adherence, as well as administrative functions [9]. In particular, within ICUs, AI is primarily employed in predictive modeling, medical imaging analysis, patient monitoring, virtual medical consultations, education and research, and clinical decision support systems [12].

### 3.1. Sepsis and Sepsis Shock

Sepsis and septic shock are one of the most important causes of death in ICU: early identification and correct management in the few hours of development of the disease improve outcomes [13,14]. MLMs analyzing datasets from electronic healthcare records or monitoring vital signs, laboratory parameters, and the medical history of patients have shown important contributions to early diagnosis, therapeutic decision support and prediction of sepsis-related death [14].

Concerning early diagnosis, in a study performed by Barton et al., an ML algorithm was developed to predict sepsis up to 48 h before onset, using six vital signs like heart rate, respiratory rate, temperature, systolic and diastolic blood pressure and oxygen saturation. The algorithm was developed on retrospective data from two different datasets and was compared with traditional scoring systems such as Sequential Organ Failure Assesment (SOFA), Modified Early Warning Score (MEWS) and quick SOFA (qSOFA) with better results [15]. Compared with conventional clinical scores such as qSOFA, SOFA, and MEWS, several machine learning models have demonstrated improved discrimination for early sepsis prediction. However, superior predictive performance does not necessarily translate into greater clinical utility. The real value of these models depends on whether earlier predictions lead to timely interventions, improve clinician decision-making, and ultimately result in better patient outcomes—questions that still require prospective implementation studies.

Lauritsen et al. proposed a model that integrated not only clinical parameters within and out of ICU, but also the treatments of the patients (intravenous antibiotics, blood culture results) recorded from multiple medical centers using a combination of two different models: convolutional neural networks (CNNs) and Long Short-Term Memory (LSTM). The results showed that early recognitions with correct therapies could reduce morbidity and mortality [16].

Although these models demonstrated strong predictive performance during development and internal validation, evidence supporting their external validation remains limited. Barton et al. developed and validated their model using retrospective datasets from two healthcare systems, whereas Lauritsen et al. trained and evaluated their deep learning model on multicenter electronic health record data. However, prospective validation across independent ICU populations and diverse healthcare settings is still scarce, limiting the generalizability of these models to routine clinical practice.

Early treatment is largely recognized as an important action to reduce mortality in sepsis but could be very complicated for the different phenotypes and variable patient response [11,12]. Fluid therapy is a cornerstone of sepsis treatment, but in some patients, aggressive fluid resuscitations show an increase in mortality and morbidity [15]. Bataille et al. demonstrated that the use of ML to collect echocardiographic data from 100 ICU patients can help identify fluid responder patients better than using clinical diagnosis (i.e., passive leg raising) [17,18]. Using another predicting model (Gradient Boosted Machine), Song et al. showed the accuracy of fluid therapy in a subgroup of patients identified using parameters such as hypotension and lactate [19].

ML methods demonstrate relatively favorable predictive performance for early mortality in sepsis and are better than some clinical scores, and can be utilized as a predictive tool for early risk stratification, as demonstrated Zhang et al. [20]. Diwan et al. developed an XGBoost-based machine learning model for predicting in-hospital mortality in patients with sepsis. SHAP (SHapley Additive exPlanations) was used as an explainability method to identify and interpret the clinical variables contributing most to the model’s predictions, thereby improving the transparency and interpretability of the algorithm rather than serving as a predictive model itself [21].

### 3.2. Acute Respiratory Distress Syndrome

The use of AI in supporting diagnosis and clinical decisions in respiratory dysfunctions is gaining increasing relevance. In particular, AI has emerged as a significant adjunct in the management of Acute Respiratory Distress Syndrome (ARDS) in the ICU. Evidence suggests that ML can be useful across multiple aspects, including diagnosis, risk stratification and prognostication [22].

AI enhances diagnostic accuracy through the advanced analysis of chest radiograph and computed tomography scans. Image-based AI models can detect diffuse bilateral opacities, quantify the extent of lung involvement, and monitor disease progression with greater speed and consistency than manual interpretation alone [22]. Notably, as highlighted by Rubulotta et al., ML algorithms have shown considerable potential for the early identification of ARDS in critically ill patients through the integrated analysis of heterogeneous clinical data, including vital signs, laboratory results, and image findings [22].

Artificial intelligence-based models have shown the ability to identify patients at risk of ARDS earlier than conventional clinical criteria. This early risk stratification may enable timely preventive strategies and optimized monitoring, potentially mitigating clinical deterioration and severe complications [23].

As regards ventilator management, recent studies have explored the application of reinforcement learning-based artificial intelligence models to support lung-protective strategies by continuously integrating physiological and clinical data. These approaches aim to assist clinicians in adapting ventilator settings, such as tidal volume and positive end-expiratory pressure, to the evolving condition of critically ill patients, potentially improving decision consistency compared with static, protocol-driven ventilation strategies [24].

Xu et al. presented an ML-based model aimed at predicting the incidence and mor-tality of ARDS in the context of nonpulmonary sepsis. The model demonstrated promising predictive performance for identifying patients at increased risk of ARDS in the context of non-pulmonary sepsis. Its potential clinical value lies in supporting earlier risk stratification and monitoring; however, evidence that its implementation improves patient management or clinical outcomes remains limited [25].

A similar perspective is presented in the study by Bai et al., which validates ML methods to predict the risk of sepsis-associated ARDS. In particular, the findings suggest that the use of AI in the early diagnosis and classification of this condition can support clinicians in making timely decisions and promoting individualized and precise treatment approaches [26].

An emerging area of research is the application of reinforcement learning (RL) to mechanical ventilation management. Unlike conventional predictive models, RL algorithms are designed to identify optimal sequential ventilator strategies using longitudinal patient data. Although early results are promising, current evidence is still largely based on retrospective datasets and offline policy evaluation, and prospective clinical validation remains limited [27].

### 3.3. Weaning

In critically ill patients, extubation failure has consistently been associated with prolonged exposure to mechanical ventilation, a higher burden of complications, and overall worse clinical outcomes. These observations underscore the limitations of current decision-making approaches and highlight the need for more comprehensive, multiparametric strategies, including the potential contribution of artificial intelligence-based predictive models, to better support extubation decisions in the ICU [28].

Recent evidence suggests that artificial intelligence and machine learning ap-proaches may support clinicians throughout the weaning process by integrating mul-timodal clinical and ventilatory data, thereby enhancing risk stratification, anticipating readiness for spontaneous breathing, and assisting extubation decision-making, while complementing rather than replacing clinical judgment [29].

Liu and colleagues designed a two-stage machine learning system to estimate the optimal timing for weaning patients from mechanical ventilation. In the first stage of the study, they modeled 25 clinical features (such as demographic data, ventilator settings and vital signs) to predict when patients might successfully transition to spontaneous or supported breathing; in the second stage, using 20 features, they predicted full weaning or extubation. By testing seven different algorithms across 11 time points for each stage, their models achieved high discriminative performance in predicting successful weaning and extubation. These findings suggest that AI-based prediction models may support clinicians in identifying patients at risk of difficult weaning and in individualizing weaning strategies. However, prospective evidence demonstrating reductions in the duration of mechanical ventilation or the risk of re-intubation remains limited [25]. These findings may be particularly relevant in patients at risk of difficult weaning, in whom repeated spontaneous breathing trials or prolonged liberation from mechanical ventilation are common. Early identification of these patients through AI-based prediction models could support a more individualized weaning strategy, although prospective evidence demonstrating improved clinical outcomes is still limited.

Similarly, Xu et al. built a risk prediction model for difficult weaning: these models aimed to help clinicians identify patients at risk for prolonged or failed weaning, facil-itating early intervention and more individualized ventilatory management [30].

To compare between two different AI models (Xboost and Random Forrest) for extubation decisions, Huang et al. compared different machine learning models for extubation decision support and reported promising predictive performance for identifying patients at risk of extubation failure. Similarly, Jiang et al. developed AI-based models to predict prolonged dependence on mechanical ventilation in patients with severe orthopedic trauma. These models may support individualized ventilatory management and resource planning, although evidence of improved patient outcomes through prospective clinical implementation is still limited [31,32].

### 3.4. Heart Failure

In patients with acute heart failure (HF), AI may assist clinicians in earlier diagnosis, hemodynamic risk assessment, and prognostic stratification. By integrating ECG findings, imaging data, laboratory results, and vital-sign trends, AI-based systems can help identify patients at higher risk of deterioration or in need of treatment escalation. These tools may also support workflow efficiency and reduce cognitive burden, although their role should remain complementary to clinical judgment [33].

### 3.5. Imaging

The integration of AI in medical imaging has significantly enhanced ICU capabili-ties, improving diagnostic accuracy, prognostic assessment, and clinical decision-making. AI algorithms accelerate the interpretation of images—such as Computed Tomography (CT) scans, MRIs, and X-rays—enabling faster detection of various critical conditions [34]. Recent advances in deep learning have enabled the integration of radiological imaging and clinical data to support predictive modeling in intensive care unit settings. For example, models that combine chest X-ray features with physiological and laboratory measurements have demonstrated improved performance in predicting ICU mortality compared with traditional scoring systems, highlighting the value of multimodal data fusion for early risk stratification and proactive patient management [35].

This reduces diagnostic delays and supports timely interventions in high-pressure ICU settings. AI has the potential to reduce inter-observer variability and support clinicians in image interpretation, potentially mitigating diagnostic fatigue and cognitive bias. However, these benefits are currently supported mainly by retrospective studies evaluating diagnostic performance, whereas prospective evidence demonstrating their impact in routine clinical practice remains limited [36].

Concerning medical imaging, AI models have demonstrated high diagnostic performance in several imaging-based diagnostic and prognostic tasks, particularly for common thoracic pathologies in critically ill patients [36]. Laino et al. evaluated a CT-based predictive model for COVID-19 that stratified patients according to imaging features such as ground-glass opacities, crazy-paving pattern, and consolidation, supporting radiological risk assessment in critically ill patients [37,38].

### 3.6. Monitoring

Continuous monitoring is a normal practice in ICU: early recognition of changes in vital signs is a cornerstone of intensive care monitoring. AI-based monitoring systems may support the earlier detection of clinical deterioration, improve the identification of false alarms, and assist clinicians in prioritizing relevant physiological changes. However, evidence demonstrating improvements in patient morbidity or mortality through prospective implementation remains limited [39,40].

Mizna et al. reported a positive impact on post-operative ICU results measuring recovery status and the presence of post-operative complications after robotic surgery through the analysis of the data trends in a patient, such as the rate of breathing, blood pressure, and blood test results [12]. Mollura et al. showed how early recognition of changes in blood pressure wave and electrocardiograms could help caregivers to make precocious diagnosis of sepsis [40]. Delirium is a strong predictor of increased length of mechanical ventilation, longer ICU stays, increased cost, long-term cognitive impairment, and mortality [41]. Davoudi et al. showed that using video monitoring systems to evaluate patients’ facial expression and using accelerometer positioning on the patient to evaluate patient movement, integrated with vital signs, could characterize delirium and patient environment [42]. A similar strategy was utilized by Wu et al. to establish a deep learning-based pain classifier based on facial expressions, acquiring video sequences and processing facial images [43]. The implementation of AI-based monitoring systems using video or wearable sensor data also raises ethical considerations regarding privacy and informed consent, particularly in ICU patients who may temporarily lack decision-making capacity. These issues are discussed in greater detail in the dedicated ethical and medico-legal section.

False alarms in the ICU, like false critical ECG arrhythmia or arterial blood pressure waveforms, generate a noisy environment and cause alarm fatigue among caregivers [44]. Using a signal quality index to distinguish noise/artifacts from normal physiological signals, Au-Yeung used an ML-based approach to assess the suppression of false alarms in real time [45]. Another important benefit of ML models was reported by Hever et al. in a study in which some parameters were deliberately removed: random forest maintained its capability to recognize false alarms in the absence of one parameter compared with full expert-based rules [46]. To minimize these risks, AI monitoring systems should be integrated into existing ICU workflows and prioritize clinically relevant alerts rather than generating excessive notifications. Explainable recommendations and the possibility for clinicians to contextualize or override AI outputs may further reduce unnecessary cognitive burden and improve usability.

### 3.7. Clinical Decision-Making

As shown in the previous sections AI can support ICU health workers in the diagnosis and treatment of many pathologies, but some contributions have also been demonstrated for mortality, risk stratification and prognosis.

In the ICU, risk stratification and the prediction of mortality is important for early intervention strategies and optimal resource allocations. Many scores are widely applied to estimate mortality risk, such as APACHE (Acute Physiology and Chronic Health Evaluation), SOFA, and the Simplified Acute Physiology Score (SAPS) [47]. Dhami et al., in a systematic review, demonstrated that ML models can improve the sensitivity and reliability of these scores for mortality prediction: the success of these results could depend on their ability to capture complex, non-linear relationships between clinical variables and mortality outcomes [48].

Because of the high mortality and incidence of disability associated with cardiac arrest and traumatic brain injury (TBI), these events have an important impact in the ICU and the prediction of prognosis is crucial to define the most appropriate therapeutic approach for the patient [49,50]. With regard to cardiac arrest patients, one retrospective study showed the accuracy of AI in the prognosis of patients by analyzing clinical and laboratory markers [51]. In patients affected by TBI, it is important to identify early predictors of patient prognosis and outcomes, as well as implementing effective treatments. Ding et al. have shown high predictive performance with ML, especially for the three clinical outcomes of interest: survival, neurological status, and length of stay in hospital [52]. This consideration is particularly important in end-of-life decision-making. Although AI may support prognostic assessment, decisions regarding the limitation or withdrawal of life-sustaining treatment should remain the result of shared decision-making based on clinical judgment, ethical deliberation, and patients’ values and preferences, with AI serving solely as a decision support tool.

### 3.8. Medico-Legal Implications in Italy

From a medico-legal perspective, the integration of artificial intelligence in intensive care represents a profound yet complex transformation, affecting not only clinical practice but also the very foundations of professional liability, patient rights protection, and healthcare governance [53,54]. Therefore, the professional liability of physicians in the use of artificial intelligence in intensive care can be understood as an extension of the traditional principles of medical negligence, in light of the technological innovations introduced. Under the Italian Law Gelli-Bianco, healthcare professionals are required to adhere to established clinical best practices and accredited guidelines, while retaining a central and non-delegable role in the decision-making process [55]. The use of AI systems does not exempt physicians from liability; rather, it imposes an additional duty of critical oversight regarding the reliability, relevance, and applicability of algorithmic recommendations to the individual clinical case. Professional negligence may arise both from uncritical reliance on machine outputs (automation bias) and from the unjustified refusal to adopt validated technological tools, thus creating a potential tension between innovation and clinical prudence. In this context, AI functions as a decision support tool lacking legal subjectivity [56,57]; consequently, ultimate responsibility for clinical decisions remains largely with the physician, both in civil and criminal domains, with potential extensions to the healthcare institution and the system manufacturer in cases of defects in the design, training, or functioning of the algorithm. Depending on the circumstances, liability may arise from clinician negligence, institutional failures in governance or oversight, defects in the design or functioning of the system, or non-compliance with applicable regulatory requirements. The adoption of such systems therefore requires a careful balance between two opposing risks: on the one hand, uncritical reliance on algorithmic recommendations; on the other, the failure to integrate technologies that may significantly improve clinical outcomes. In intensive care, AI has shown promising predictive performance in several intensive care applications, including early sepsis detection, cardiac arrest prognostication, optimization of mechanical ventilation, and prediction of mortality or readmission. Several models have outperformed traditional clinical scoring systems in retrospective validation studies; however, evidence demonstrating improved patient-centered outcomes following routine clinical implementation remains limited [58,59], often outperforming traditional clinical scoring systems and contributing to a possible reduction in length of stay through the real-time analysis of multimodal data. However, these benefits are accompanied by significant challenges. A central issue concerns decision-making transparency: many AI systems operate as “black boxes,” making it difficult to reconstruct and reproduce the logical process leading to a given recommendation [60]. This opacity may undermine both clinical trust and the defensibility of decisions in legal settings, exposing healthcare professionals to substantial risks in terms of professional liability. This responsibility becomes particularly challenging when AI systems operate as black box models, as clinicians may not be able to fully evaluate the reasoning underlying algorithmic recommendations, reinforcing the need for appropriate human oversight and institutional governance.

For this reason, increasing importance is being placed on the development and adoption of explainable AI models [61,62], capable of ensuring traceability, interpretability, and proper documentation of the decision-making process.

Another critical issue involves algorithmic bias. Non-representative datasets may lead to systematic errors and ethically significant discrimination against vulnerable groups, such as ethnic minorities, older adults, or individuals with lower socioeconomic status, thereby violating the principle of equity. For instance, some studies have shown that AI systems trained predominantly on data from Caucasian populations may exhibit reduced reliability when applied to other ethnic groups [63]. Such distortions, particularly relevant in contexts like intensive care imaging, necessitate the implementation of pre-deployment audits, data diversification strategies, and fairness testing in order to mitigate the risk of liability also from an anti-discrimination perspective.

Algorithmic bias may also arise when AI models are developed using datasets that are not representative of the broader ICU population. For example, models trained on single-center or geographically homogeneous datasets may show reduced performance when applied to different patient populations, underscoring the importance of external validation and diverse training datasets [64].

From a regulatory standpoint, the European and national frameworks are rapidly evolving. Data protection legislation imposes strict requirements on the management of clinical data, including high standards of security, anonymization, and lawfulness of processing, as well as limitations on fully automated decision-making without human oversight [65]. In parallel, recent European regulation on artificial intelligence classifies the majority of systems used in healthcare, and particularly in intensive care, as high risk, requiring rigorous conformity assessment procedures, risk management, transparency, and continuous monitoring. Under the EU AI Act, the classification depends on the specific system, its intended purpose, and the applicable provisions. AI systems used for clinical decision support in intensive care are generally considered high-risk medical AI systems and must comply with requirements for transparency, data governance, accuracy, and human oversight. Similarly, Article 22 of the GDPR reinforces that AI should support, rather than replace, clinician decision-making by ensuring meaningful human involvement in decisions with significant clinical consequences.

In this context, informed consent also assumes distinctive features: in intensive care it is often deferred or provided by a surrogate decision-maker, making it necessary to develop clear and accessible institutional forms of information regarding the use of AI, in order to ensure respect for patient autonomy as in all other areas of medicine [66].

Ultimately, the introduction of artificial intelligence in intensive care cannot be regarded solely as a technological or clinical issue, but must be addressed as an integrated matter of responsibility, safety, and ethics. A truly sustainable approach requires continuous professional training, the development of shared guidelines, oversight by ethics committees, periodic audits, and appropriately tailored insurance coverage [67]. Only through a robust and multidisciplinary governance framework will it be possible to achieve a form of medicine not replaced by machines, but made safer and more effective through them, capable of combining innovation, equity, and the centrality of human responsibility [68,69].

## 4. Discussion

This narrative review underscores that, despite the rapid growth of AI in critical care, these systems should be viewed as clinical decision support tools rather than autonomous agents. While AI excels at processing vast, complex datasets and shows high predictive accuracy, there is still a lack of definitive evidence proving it improves “hard” outcomes, such as patient survival. Furthermore, moving AI from the lab to the bedside is hindered by challenges regarding algorithmic bias, limited adaptability to different hospitals, “black-box” interpretability, and complex legal questions.

Most AI models perform exceptionally well in retrospective tests, often outperforming traditional clinical scoring systems [70]. However, high statistical accuracy does not automatically lead to better healthcare [71]. There is a critical need to bridge the gap between a model’s ability to predict an event and its proven ability to benefit the patient in a real-world setting [72]. Most existing models are trained on data derived from single centers or narrowly defined patient populations, which limits their reliability when applied to different clinical settings [73].

Variations in patient characteristics, case mix, and local clinical practices can substantially affect model performance once deployed beyond the original development environment. Consequently, robust external validation and continuous performance monitoring are critical to ensure safe and effective implementation [74,75,76,77,78].

AI systems can inadvertently “learn” and amplify or systemic biases found in their training data, which could lead to unfair treatment of underrepresented groups [79,80,81]. To ensure ethical care, we must rigorously audit data for representativeness and ensure that algorithms do not worsen existing healthcare disparities [82,83,84]. A major hurdle to AI adoption is the “black box” nature of complex algorithms. If clinicians cannot understand the reasoning behind a recommendation, they are unlikely to trust it [84,85,86].

Artificial intelligence should be regarded as a clinical decision support technology rather than a substitute for physician judgment or direct patient evaluation. In intensive care, where clinical decisions rely on continuous bedside assessment and contextual interpretation, AI outputs must always be integrated with physical examination, clinical expertise, and multidisciplinary decision-making. Furthermore, generative AI systems may produce inaccurate or fabricated information (“hallucinations”), particularly when operating outside validated clinical contexts. If such information is interpreted as reliable medical advice, it may contribute to inappropriate health decisions, delayed medical evaluation, or the spread of misinformation. These risks reinforce the need for human oversight, transparent communication, and the use of validated AI systems in healthcare [10].

These considerations are particularly relevant because patients and caregivers increasingly access health information through AI tools and online platforms, where information quality and scientific reliability are highly variable.

While “Explainable AI” (XAI) is emerging, it often only provides simplified approximations of the logic [87]. Therefore, human oversight remains indispensable to ensure AI outputs are interpreted within the correct clinical context [88,89]. The use of AI in life-or-death situations raises difficult questions about who is at fault when something goes wrong [90]. Some authors also suggest that artificial intelligence may support end-of-life care by improving prognostication and organizing previously expressed patient preferences. However, limitations in reliability, reproducibility, fairness, and—most importantly—the absence of empathy and contextual moral judgment fundamentally constrain AI’s role in ethical decision-making, where human values cannot be reduced to computational logic [91].

Currently, the clinician is generally seen as the final decision-maker, while institutions and developers handle system governance. Clearly defined legal frameworks are needed to manage responsibility and ensure transparency. New regulations, such as the EU-AIA, categorize healthcare AI as “high-risk,” requiring strict safety and transparency standards. However, there is still a lack of practical, day-to-day guidance on how to implement these rules in a clinical workflow. Global standards are needed to harmonize these processes [92]. For AI to be effective, it must fit seamlessly into the busy ICU environment. Poorly integrated tools risk overwhelmed staff with “alert fatigue” or adding to their cognitive load. AI should provide brief, actionable insights that enhance, rather than interrupt, a doctor’s natural decision-making process.

The allocation of responsibility for AI-assisted clinical decisions is more complex than attributing liability exclusively to the treating physician. While clinicians remain responsible for integrating AI recommendations into the overall clinical assessment and final decision-making process, responsibility may also involve healthcare institutions implementing AI systems, developers and manufacturers of AI-based medical devices, and the regulatory framework governing their approval and post-market surveillance. The extent of accountability depends on the intended use of the AI system, the degree of human oversight, and compliance with applicable regulatory requirements. Therefore, AI should be regarded as a decision support technology operating within a framework of shared professional, institutional, and regulatory responsibilities rather than as an autonomous clinical decision-maker [7].

### 4.1. Future Directions

Future clinical evaluation should prioritize prospective multicenter implementation studies using pragmatic trial designs, such as cluster randomized or stepped-wedge trials, which are well suited to evaluating AI tools integrated into ICU workflows. In addition to predictive accuracy, these studies should assess clinically meaningful outcomes, including mortality, duration of mechanical ventilation, ICU length of stay, time to therapeutic intervention, and clinician workload or alert fatigue [32]. Improving external validity will require multicenter prospective validation studies and the inclusion of geographically and demographically diverse ICU populations. Federated learning represents a promising strategy by enabling collaborative model development across institutions without sharing sensitive patient-level data, potentially improving model generalizability while preserving data privacy [64].

Across the clinical applications discussed in this review, a consistent pattern emerges: AI models frequently demonstrate strong predictive performance during model development and internal validation, but substantially fewer systems have undergone external validation or prospective clinical implementation. Differences in patient populations, clinical workflows, data quality, and electronic health record structures may reduce model performance when algorithms are transferred across institutions. This translational gap highlights the fact that successful implementation in intensive care depends not only on algorithmic accuracy, but also on external validation, workflow integration, clinician oversight, interpretability, and continuous post-implementation monitoring.

### 4.2. Strengths of the Review

The main strength of this narrative review is its interdisciplinary perspective, integrating current clinical applications of artificial intelligence into intensive care with ethical, regulatory, and medico-legal considerations. Rather than focusing on a single clinical domain, this review discusses the opportunities and implementation challenges of AI across critical care practice, emphasizing the gap between predictive performance and real-world clinical impact. This integrated approach aims to support clinicians in interpreting AI as a decision support technology within a patient-centered and ethically responsible framework [92].

### 4.3. Limitations

This narrative review has several limitations that should be acknowledged. First, the non-systematic design of the literature search may have resulted in the omission of relevant studies, and no formal quality assessment or quantitative synthesis was performed. As a consequence, the findings reflect a qualitative interpretation of the available evidence rather than a comprehensive or exhaustive appraisal. Second, the evidence discussed is largely derived from retrospective and observational studies, many of which rely on single-center datasets and lack external validation. This limits the strength of inferences regarding clinical effectiveness and generalizability across different ICU settings. Third, the rapidly evolving nature of artificial intelligence in critical care implies that some technologies and regulatory frameworks discussed may change over time, potentially affecting the long-term applicability of the conclusions. In addition, publication bias toward positive or high-performing AI models cannot be excluded.

Finally, given the breadth of the topic, this review focused on selected high-impact use cases and ethical–regulatory aspects, rather than providing a detailed technical comparison of algorithms or performance metrics. This pragmatic approach was chosen to emphasize clinical relevance and translational considerations but may have limited the depth of analysis in specific methodological domains.

## 5. Conclusions

Artificial intelligence holds significant promise in intensive care, with applications ranging from early diagnosis to risk stratification and decision support. However, despite strong predictive performance, robust evidence demonstrating improvement in patient-centered outcomes remains limited, and issues of generalizability, bias, and interpretability persist. The integration of AI into clinical practice also raises critical ethical and medico-legal challenges, particularly regarding transparency, accountability, and data governance. In this context, regulatory frameworks and explainable models are essential to ensure safe and equitable use.

As access to AI-generated health information through online platforms continues to expand, these technologies should be considered supportive educational tools rather than primary sources for patient information or independent instruments for clinical decision-making. Information generated by AI or distributed through online platforms may lack the depth required for individualized risk–benefit assessment, recognition of warning signs, and application of evidence-based clinical guidelines. Therefore, AI-supported information must always be interpreted within the context of professional medical evaluation and shared clinical decision-making [11].

Ultimately, AI should be considered a support tool rather than a replacement for clinical judgment. Its successful implementation will depend on rigorous validation, thoughtful integration into clinical workflows, and close collaboration between clinicians, researchers, and policymakers to ensure that innovation remains aligned with patient care and ethical principles.

## Data Availability

No new data were created or analyzed in this study. Data sharing is not applicable to this article.

## Abbreviations

The following abbreviations are used in this manuscript:

AI	Artificial Intelligence
ICU	Intensive Care Unit
GDPR	General Data Protecting Regulation
ML	Machine Learning
LLMS	Large Language Models
DL	Deep Learning
AIA	Artificial Intelligence Act
MLMs	Machine Learning Models
SOFA	Sequential Organ Failure Assesment
SIRS	Systemic Inflammatory Response Syndrome
MEWS	Modified Early Warning Score
qSOFA	Quick Sequential Organ Failure Assesment
CNNs	Convolutional Neural Networks
LSTM	Long Short-Term Memory
ARDS	Acute Respiratory Distress System
HF	Heart Failure
ECG	Electrocardiogram
BUN	Blood Urea Nitrogen
CT	Computed Tomography
GGO	Ground Glass Opacities
APACHE	Acute Physiology and Chronic Health Evaluation
SAPS	Simplified Acute Physiology Score
TBI	Traumatic Brain Injury
XAI	Explainable AI
